# A New Strategy for Fast MRI-Based Quantification of the Myelin Water Fraction: Application to Brain Imaging in Infants

**DOI:** 10.1371/journal.pone.0163143

**Published:** 2016-10-13

**Authors:** Sofya Kulikova, Lucie Hertz-Pannier, Ghislaine Dehaene-Lambertz, Cyril Poupon, Jessica Dubois

**Affiliations:** 1 INSERM U1129, CEA/DRF/I2BM/Neurospin/UNIACT, Gif-sur-Yvette, France; Université Paris-Saclay, Université Paris Descartes, Sorbonne Paris Cité, Paris, France; 2 INSERM U992, CEA/DRF/I2BM/Neurospin/UNICOG, Gif-sur-Yvette, France; Université Paris Saclay, Université Paris-Sud, Gif-sur-Yvette, France; 3 CEA/DRF/I2BM/Neurospin/UNIRS, Gif-sur-Yvette, France; Université Paris Saclay, Université Paris-Sud, Gif-sur-Yvette, France; Karl-Franzens-Universitat Graz, AUSTRIA

## Abstract

The volume fraction of water related to myelin (*f*_*my*_) is a promising MRI index for *in vivo* assessment of brain myelination, that can be derived from multi-component analysis of T1 and T2 relaxometry signals. However, existing quantification methods require rather long acquisition and/or post-processing times, making implementation difficult both in research studies on healthy unsedated children and in clinical examinations. The goal of this work was to propose a novel strategy for *f*_*my*_ quantification within acceptable acquisition and post-processing times. Our approach is based on a 3-compartment model (myelin-related water, intra/extra-cellular water and unrestricted water), and uses calibrated values of inherent relaxation times (*T*1_*c*_ and *T*2_*c*_) for each compartment *c*. Calibration was first performed on adult relaxometry datasets (N = 3) acquired with large numbers of inversion times (TI) and echo times (TE), using an original combination of a region contraction approach and a non-negative least-square (NNLS) algorithm. This strategy was compared with voxel-wise fitting, and showed robust estimation of *T*1_*c*_ and *T*2_*c*_. The accuracy of *f*_*my*_ calculations depending on multiple factors was investigated using simulated data. In the testing stage, our strategy enabled fast *f*_*my*_ mapping, based on relaxometry datasets acquired with reduced TI and TE numbers (acquisition <6 min), and analyzed with NNLS algorithm (post-processing <5min). In adults (N = 13, mean age 22.4±1.6 years), *f*_*my*_ maps showed variability across white matter regions, in agreement with previous studies. In healthy infants (N = 18, aged 3 to 34 weeks), asynchronous changes in *f*_*my*_ values were demonstrated across bundles, confirming the well-known progression of myelination.

## Introduction

Myelination is a crucial process of the white matter maturation. Although there is a critical need for *in vivo* quantitative assessment of myelination in many clinical conditions encompassing most neurodevelopmental disorders, there is still no gold-standard for its evaluation. Whereas myelination can be assessed using conventional Magnetic Resonance Imaging (MRI) and Diffusion Tensor Imaging (DTI) parameters [1–3], these methods provide only indirect and non-specific measures of the myelin content as they also reflect other tissue properties [4,5]. Recent advances in MR relaxometry have proposed a novel index: the volume fraction of water related to myelin (*f*_*my*_), which has shown good correlation with the myelin amount [6] and seems relevant for quantifying the progression of white matter myelination [7–9]. *f*_*my*_ corresponds to the fraction of water volume trapped by the myelin sheaths relative to the total water volume within an imaging voxel, and can be derived from a multi-component analysis of relaxometry signals [10,11]. The number of compartments, corresponding to different pools of water within a voxel, usually varies from 2 to 4, and includes myelin-related compartment, intra/extra-cellular water, and unrestricted water (such as observed in the cerebro-spinal fluid CSF).

The most conventional approaches for *f*_*my*_ quantification rely on the calculation of T2 spectrum, and define *f*_*my*_ in each voxel as the ratio between the signal with T2 below 40-50ms, and the total water signal [6,11–16]. Similar strategies have been proposed for T2* [17] and T1 spectra [18]. However, reliable estimation of a spectrum requires acquisition of relaxometry signals with a large number of sampling points (N>32 for echo times TE or inversion times TI) [10,11], making the acquisition protocol impractically long for unsedated infants and children. Alternative approaches are based on multi-compartment models of MR relaxometry signals, which take into account the relative contributions of different compartments: fitting these models against acquired measurements enables to estimate the volume fractions of the various compartments [9,19,20]. In such models, differences in relaxometry signals across voxels might be explained either by different compartment fractions, or by variations in their magnetic properties (i.e. intrinsic relaxation times) due to variations in their biophysical properties (axonal diameters, myelin thickness, myelin compactness, axonal density, etc.). Unless *a priori* assumptions are made on the compartment relaxation times [9], such models are described by non-linear equations, and the robustness of their fitting relies on the sampling strategy, the number of measurements and the optimization algorithm. mcDESPOT sequences were the first approach that allowed to reduce the acquisition time: for instance, in infants between 3 and 9 months of age, the total imaging time (including sequences for B_0_ and B_1_ corrections [21]) can be reduced down to 18min on a 3T scanner [8]. Unfortunately this duration remains rather long for unsedated pediatric subjects. In addition, long post-processing times (~14 hours per subject on an 8-core Intel I7 machine [19]) are inevitable due to the model non-linearity imposing the use of time-consuming stochastic model fitting. The goal of this study was thus to design and test a novel strategy for fast *f*_*my*_ quantification, based on reasonable acquisition and post-processing times, which could be applied for instance in infants.

## Strategy and Hypotheses for Fast *f*_*my*_ Quantification

Let us first introduce the general concepts and hypotheses of our approach. It is based on a 3-compartment relaxation model adapted from [9], and uses a 2-stage strategy (calibration then testing), which imposes little *a priori* assumptions on the compartment relaxation properties. Details on the fitting procedures are provided in the “Materials and Methods” section.

### A 3-Compartment Model

A compartmental model aims to decompose the measured NMR signals at the voxel level into components characterized by different relaxation times corresponding to different intra-voxel compartments [19,22–24]. The variability in measured signals observed across voxels could then be explained by differences in the relative compartments’ fractions within those voxels. Within each voxel, we considered three compartments with their own inherent relaxation times: myelin-related water, intra/extra-cellular water, and unrestricted water (further referred to as CSF compartment). The 3-compartment model (Eq 1–4) links the volume fractions (*f*_*c*_) of compartments (*c*) and their inherent relaxation times (*T*1_*c*_ and *T*2_*c*_), with the T1-weighted measurements (S_T1_) acquired for N_TI_ inversion times (*TI*_*m*_) and the T2-weighted measurements (S_T2_) acquired for *N*_*TE*_ echo times (*TE*_*k*_).

{∑c=13fc=1, fmy∈[0,fmyMAX], fie,csf∈[0,100%] (1);ST1(TIm)=S0(1−2exp(−TImT1)), m=1..NTI (2);1T1=∑c=13fcT1c (3);ST2(TEk)=S0∑c=13fcexp(−TEkT2c),k=1..NTE (4);

The first equation of this model merely states that there are only 3 compartments (my—myelin-related water, ie—intra/extra-cellular water, and unrestricted water compartment), with fractions varying from 0 to *f*_*myMAX*_ or 100% [9,19,25–27]. Eq 2 describes the classical relationship between T1 relaxometry signal, inversion and relaxation times. Eq 3 assumes a “fast exchange” model of T1-weighted signal: between-compartment water mixing times are assumed to be short compared with within-compartment T1 relaxation times. Eq 4 assumes a “slow exchange” model of T2-weighted signal: T2 relaxation times are assumed to be short compared with the time it takes water molecules to exchange between compartments. The validity of this model assumptions was verified in [9].

Fitting such a non-linear 3-compartment model (Eq 1–4) with a limited number of measurements is an ill-posed problem. However, if we pre-calculate *T*1 using Eq 2, and fix *T*1_*c*_ and *T*2_*c*_ for each compartment *c* in Eq 3, only 3 parameters are unknown (i.e. the compartment fractions *f*_*c*_), and the model becomes linear. Such a model can be easily fitted based on a reasonable number of measurements and using standard non-negative least-square (NNLS) algorithms [28].

### A 2-Stage Strategy

In the adult brain, some variability in T1 and T2-weighted signals measured at the voxel level is observed across brain tissues and regions. Here we assume that this variability can be fully explained by differences in the volume fractions of compartments, while *T*1_*c*_ and *T*2_*c*_ for each compartment *c* can be considered constant across voxels and subjects as they characterize the tissue magnetic properties. In this context, we implemented the following 2-stage strategy to reliably estimate *f*_*my*_ maps. First, because *T*1_*c*_ and *T*2_*c*_ might depend on the acquisition protocol (B_0_ field, sequences, etc.), a dedicated calibration procedure became mandatory. To this aim, we used datasets acquired in healthy adults with a large number of measurements, to compute the *T*1_*c*_ and *T*2_*c*_ values and ensure a robust fit of the full 3-compartment relaxation times (Eq 1–4) to be used next. This “calibration stage” was achieved by an original combination of a region contraction approach and a standard NNLS algorithm. Second, the model complexity was reduced by fixing *T*1_*c*_ and *T*2_*c*_ in the “testing stage”, which allowed us to compute *f*_*my*_ maps from datasets acquired using a significantly reduced imaging protocol relying on a small number of measurements. To check the validity of our hypothesis, we compared *f*_*my*_, *T*1_*c*_ and *T*2_*c*_ values computed with voxel-wise model fitting, and at the calibration and testing stages. The impact of several factors on *f*_*my*_ accuracy was also investigated.

Besides, compartmental models have enabled the efficient description of changes related to brain maturation in the recent years [8,9,29]. Here we evaluated whether *f*_*my*_ maps could be computed in infants with the same approach, based on reduced datasets relying on few measurements. As detailed in the “Discussion” section, we assumed that *T*1_*c*_ and *T*2_*c*_ do not evolve throughout development, and that age-related decreases in T1 and T2 relaxation times measured at the voxel level are exclusively explained by age-related changes in intra-voxel compartments fractions.

## Materials and Methods

### Datasets and MRI Acquisition

We acquired different datasets in adult and infant subjects. All acquisitions were performed on a 3T MRI system (Tim Trio, Siemens Healthcare, Erlangen, Germany), equipped with a whole body gradient (40mT/m, 175T/m/s) and a 32-channel receive-only head coil. To measure T1 and T2 relaxometry signals, single-shot spin-echo (SE) echo-planar-imaging (EPI) sequences were used to acquire interleaved axial slices covering the whole brain (70/50 slices for adults/infants) with a 1.8 mm isotropic spatial resolution (field-of-view = 23cm, matrix = 128x128, slice thickness = 1.8mm). For T1 relaxometry, an inversion recovery (IR) SE-EPI sequence with different TI values was used (TE = 38ms, TR = TI + 21s/15s for adults/infants; partial Fourier sampling factor of 5/8). For T2 relaxometry, a SE-EPI sequence with different TE values was used (TR = 21.7s/15.5s for adults/infants, parallel imaging with GRAPPA factor 2, partial Fourier sampling factor of 6/8). Anatomical images were further acquired for registration: in adults, T1-weighted (T1w) images with a 1mm isotropic spatial resolution using a 3D fast gradient inversion recovery sequence (MPRage); in infants, T2-weighted (T2w) images with a spatial resolution of 1x1x1.1mm^3^ using a 2D turbo spin echo sequence.

#### Calibration datasets in adults

To calibrate *T*1_*c*_ and *T*2_*c*_ of each compartment *c*, we acquired data on 3 healthy adult volunteers (1 female, 2 males, mean age: 23.2±2 years) using long protocols, based on large numbers (N = 30–60) of TI (sampled between 100ms and 3100ms) and TE (sampled between 30ms and 340ms) leading to acquisition times between 35 and 47min (S1 Table and S1 Fig). The TI and TE samplings were denser at the beginning of the sampling range to ensure good parameters estimation for the fast-relaxing myelin-related compartment with low *T*1_*my*_ and *T*2_*my*_.

#### Reduced datasets in adults and infants

The strategy was tested in two groups using short acquisition protocols: an adult group of 13 healthy subjects (6 females, 7 males, mean age: 22.4±1.6 years), and a group of 18 healthy infants born at term (8 girls, 10 boys), with a maturational age (i.e. chronological age corrected for gestational age at birth) between 3 and 21 weeks for 17 infants, plus one infant of 34 weeks old. A reduced number of measurements was acquired. For T1 relaxometry, 8 different TIs (from 250 to 1500ms every 250ms, 2000 and 2500ms) were used, leading to a scan duration of 3min03s/2min11s (adults/infants). For T2 relaxometry, 8 linearly spaced TE values were used between TE = 50 and 260ms, leading to a scan duration of 4min/2min51s. To identify different white matter bundles used as regions-of-interest for *f*_*my*_ quantification, a non-diffusion weighted image (b = 0 s/mm2) and 30 diffusion-weighted (DW) images (b = 700 s/mm2) were acquired using a DW-SE-EPI sequence (TE = 72ms, TR = 14s/10s, parallel imaging GRAPPA factor 2, partial Fourier sampling factor 6/8) within an acquisition time of 7min56s/5min40s.

The study protocol was approved by the regional ethical committee for biomedical research from Kremlin-Bicêtre (for adult experiments: CPP #2008-A00241-54; for infant experiments: INSERM 123 011, CPP #05 14). All parents and adult subjects gave written informed consents. The infants were spontaneously asleep during MR imaging (the protocol length was sufficiently short to be acquired during the nap). Particular precautions were taken to minimize noise exposure, by using customized headphones and covering the magnet tunnel with special noise protection foam (“plastison”, Serenata, http://www.serenata.tm.fr/product.php?id_product=17). We also restrained the slew rate of MR gradients in EPI acquisitions. To reduce motion, the infants’ heads were gently restricted by the headphones and MRI foam pads.

### Data Post-Processing

#### Data preparation

All data were pre- and post-processed using PTK toolkit and Connectomist software both developed in-house at NeuroSpin [30,31]. For T1 and T2 relaxometry images, the signal-to-noise ratio (SNR) was computed as the ratio between the mean signal from the brain (without ventricles) to the standard deviation of the noise from the image background. For all TIs and TEs, the SNR of T1 and T2 relaxation images was above 5.3 and 6.2 respectively. These noise levels allowed us to approximate the non-centered chi-noise present in GRAPPA-reconstructed images by a Gaussian noise, and thus to use NNLS estimators.

In each subject, T1 and T2 relaxometry images were co-registered with anatomical images using affine transformations maximizing their mutual information. A brain mask was computed from the T2-weighted images with the highest SNR (corresponding to the lowest TE), based on thresholding and mathematical morphology tools (combination of opening and closing). All images were masked with this brain mask. Quantitative T1 and T2 maps were further computed.

#### Computation of reference *f*_*my*_ maps

**Fig 1:**
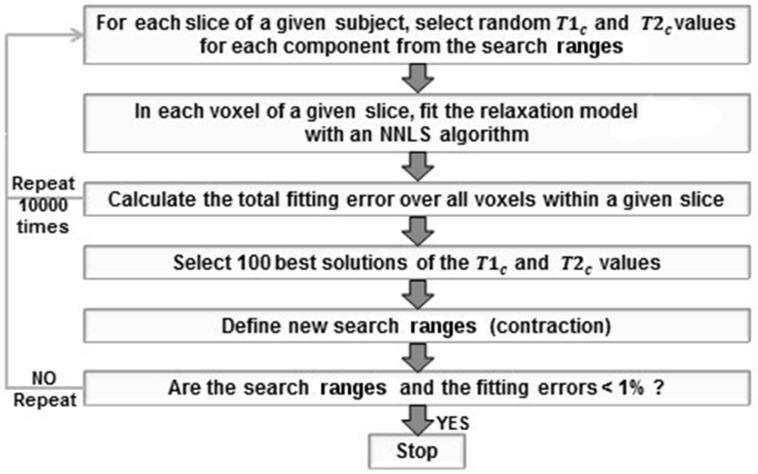
Schematic representation of the fitting algorithm. The fitting procedure was performed either for each voxel (voxel-wise approach) or for each slice (calibration approach) independently. The fitting steps were repeated until both the fitting error and the differences between the upper and lower borders of the search ranges became less than 1%.

In the calibration datasets (3 adults), we first performed a voxel-wise fitting of the 3-compartment model (Eq 1–4), to estimate *f*_*c*_, *T*1_*c*_ and *T*2_*c*_ for each compartment *c* in each brain voxel. We aimed to both ensure that there was no particular regional dependence of *T*1_*c*_ and *T*2_*c*_, and to compute reference *f*_*my*_ maps. The upper boundary for *f*_*my*_ was set to 40%. An original combination of a region contraction approach [19,32] and a standard NNLS algorithm [28] was used (Fig 1). This procedure was programmed in Python using NumPy and SciPy libraries. The initial search ranges for the individual compartment *T*1_*c*_ and *T*2_*c*_ were set based on literature evidence [9,19,33,34] and to ensure their continuity:

- for myelin-related compartment: *T*1_*my*_ ∈ [300; 570] ms, *T*2_*my*_ ∈ [1; 40] ms
- for intra/extra-cellular water compartment: *T*1_*ie*_ ∈ [570; 1600] ms, *T*2_*ie*_ ∈ [40; 200] ms
- for CSF compartment: *T*1_*csf*_ ∈ [1600; 4000] ms, *T*2_*csf*_ ∈ [200; 2000] ms.

In each voxel, random *T*1_*c*_ and *T*2_*c*_ values were first selected from the corresponding search ranges using uniform random distributions. Then the simplified model (with corresponding *T*1_*c*_ and *T*2_*c*_) was fitted using a NNLS algorithm. The fitting error was calculated by increasing the weight of Eq 3 (describing T1 relaxation) proportionally to the number of TI points: this was performed to balance the contributions of T1 (only one equation in the model) and T2 (TE equations) in the fitting error computation. Similar to [19], this step was repeated 10000 times in each voxel, and the best 100 selections of *T*1_*c*_ and *T*2_*c*_ values (i.e. providing the smallest fitting errors) were used to contract the search ranges. After contraction, the whole procedure was re-started: 90% of random *T*1_*c*_ and *T*2_*c*_ values were chosen from the new search ranges, while the remaining 10% were selected from the initial ranges to avoid falling into a local minimum. This fitting procedure was executed until both the fitting errors and the difference between the ranges boundaries became less than 1%. For each voxel, this procedure took around 70-80sec on Intel I3 machine using a single core CPU (the algorithm convergence is illustrated in S2 Fig), leading to around 120 hours per subject for the central slices of the brain (covering the ventricles, corpus callosum and basal ganglia, i.e. containing portions of all three compartments).

At the same time, *f*_*my*_ maps were computed using Eq 1–4 by fixing *T*1_*c*_ and *T*2_*c*_ based on the literature: either according to [9] (*T*1_*my*_ = 350*ms*, *T*1_*ie*_ = 850*ms*, *T*1_*csf*_ = 2800*ms*, *T*2_*my*_ = 10*ms*, *T*2_*ie*_ = 40*ms*, *T*2_*csf*_ = 130*ms*) or [19] (*T*1_*my*_ = 465*ms*, *T*1_*ie*_ = 9665*ms*, *T*1_*csf*_ = 3500*ms*, *T*2_*my*_ = 12*ms*, *T*2_*ie*_ = 90*ms*, *T*2_*csf*_ = 250*ms*). The resulting *f*_*my*_ maps were compared with maps obtained with voxel-wise fitting, with the aim to demonstrate that *T*1_*c*_ and *T*2_*c*_ values could not be simply taken from the literature but required specific calibration for our acquisition protocol.

#### Calibration of the 3-compartment relaxation model

*T*1_*c*_ and *T*2_*c*_ were then calibrated on the same datasets, using the same fitting procedure as for the voxel-wise estimation, except that it was performed at the slice level and not at the voxel level for two reasons: to save computation time, and to increase the fitting accuracy (as detailed below, calculation errors decrease with the number of voxels used for fitting). In each of the 3 subjects, we considered the same 10 central slices as they contained portions of all three compartments. The total fitting error was calculated over all voxels within each slice independently (averaged number per slice: 5718±406). Once the calibration was performed for each slice, *T*1_*c*_ and *T*2_*c*_ were set to the weighted averages over all slices of all subjects (10 slices x 3 subjects, weighted with inversed fitting errors). These calibrated values were compared with *T*1_*c*_ and *T*2_*c*_ distributions obtained with the voxel-wise approach. The resulting *f*_*my*_ maps were also compared with the reference *f*_*my*_ maps. This calibration stage took approximately 24 hours per subject.

### Checking the Accuracy and Limits of the Calibration Stage

#### Selection of *f*_*my*_ upper boundary

We explored the effect of *f*_*my*_ upper boundary at the calibration stage, since it was shown to have a considerable impact on *f*_*my*_ values derived from the relaxometry model based on mcDESPOT protocol [35]. It has been shown that the quality of model fitting (sum-of-square of residuals) using stochastic region contraction approach is the best when the upper boundary is between 0.3 and 0.5 and is insensitive to changes within this range [35]. Thus the search interval for *f*_*my*_ was initially set to [0; 0.4], and we also considered two other upper boundaries (0.3 and 0.5) at the calibration stage, to compare the estimated *T*1_*c*_ and *T*2_*c*_, and *f*_*my*_ maps. Given the results, the upper boundary was fixed to 0.4 in the following tests.

#### Impact of TI/TE numbers on the calibration of T1_c_ and T2_c_

We investigated the impact of TI and TE numbers on the estimation of *T*1_*c*_ and *T*2_*c*_, to make sure that each calibration dataset had enough sampling points for reliable calibration. From the initial dataset with 60 TI/TE sampling points (subject #3, S1 Fig), we progressively removed data points in a regular manner from 60 down to 10 points (S1 Fig), and we performed independent calibrations for each resulting dataset.

#### Evaluating the fitting accuracy using simulated data

We also checked using simulated data whether the proposed calibration strategy provided reliable computation of *f*_*my*_ values. T1 and T2 relaxometry signals were simulated using Eq 2–4 at all TI and TE sampling points. We fixed *T*1_*c*_ and *T*2_*c*_ values either equal to those identified at the calibration stage or as in previous studies [9,19,34,35] (*T*1_*my*_ = 465*ms*, *T*1_*ie*_ = 965*ms*, *T*1_*csf*_ = 3500*ms*, *T*2_*my*_ = 12*ms*, *T*2_*ie*_ = 90*ms*, *T*2_*csf*_ = 250*ms*). Different compartment fractions were considered, as possibly observed in the white matter: *f*_*my*_ = [5,10,15,20,25,30,40]%, *f*_*csf*_ = [0,1,2,3,4,5]% and *f*_*ie*_ = (100 − *f*_*my*_ − *f*_*csf*_)%. Simulated signals were additionally corrupted with a Gaussian noise with a dispersion varying from 0 to 20% relative to signal values. Then the calibration procedure was applied to simulated signals to identify the compartment fractions as well as *T*1_*c*_ and *T*2_*c*_. The error in *f*_*my*_ calculation was estimated as the averaged absolute percent difference between identified and simulated values. We also investigated how the number of voxels used for the model fitting impacted *f*_*my*_ errors. In theory, calculation errors are proportional to the inversed square root of the voxel number [36]. Thus, simulations were performed for voxel numbers equal to [1^2^, 2^2^, …, 75^2^ = 5625], and errors were fitted with the inverse square root function. Finally, we compared *f*_*my*_ calculation errors obtained in simulations using both T1 and T2 relaxometry signals together, vs. only T2 relaxometry signal.

### Testing the Calibration Model and Measuring *f*_*my*_ over White Matter Bundles

In the testing stage, *f*_*my*_ maps were computed in both the calibration and reduced datasets by fixing *T*1_*c*_ and *T*2_*c*_ to those determined at the calibration stage. For the whole brain of each subject, this stage took less than 5 min. We checked that down-sampling the calibration datasets (to match the 8 TIs and 8 TEs of the reduced datasets) had no significant impact on the generated *f*_*my*_ maps. Similarity between *f*_*my*_ distributions computed from the adult calibration and reduced datasets was assessed by ad-hoc *χ*^2^ test [37]. For each adult and infant reduced dataset, *f*_*my*_ was further quantified and averaged over different white matter bundles. To do so, DW images were co-registered with anatomical images using affine transformations, and corrected for motion and eddy current artifacts [38]. 18 projection, association, commissural and limbic bundles maturing at different times and rates were reconstructed in each subject according to a 4-order analytical Q-ball model [39], regularized 3D tractography [40], and manually delineated regions of interest [41]. In the adult group, *f*_*my*_ value in each bundle was compared with quantitative T1 and T2 relaxation times based on correlation across subjects. In the infant group, *f*_*my*_ age-related changes were assessed. Infant values were further normalized by the corresponding average from the adult group to address the degree of bundles myelination relatively to the adult mature stage [42].

## Results

### Calibration vs Voxel-Wise Fitting Approach

**Fig 2:**
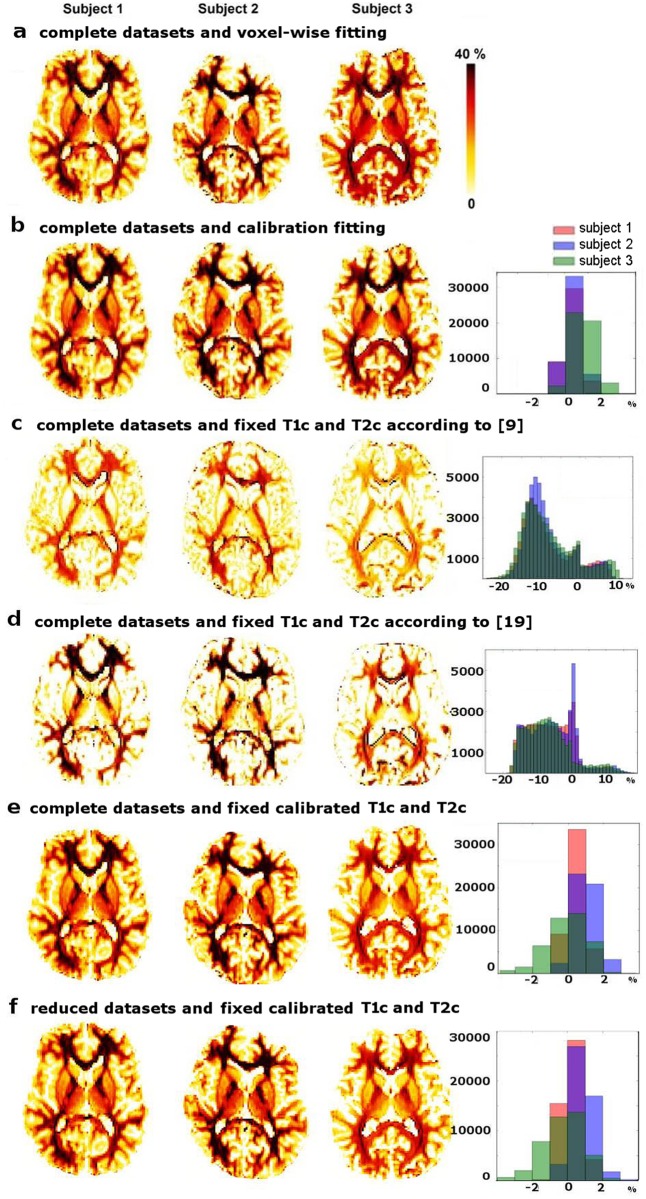
*f*_*my*_ maps in the 3 calibration adult subjects. For each subject, *f*_*my*_ maps were computed according to different strategies based on either the complete datasets (a-e) or datasets with reduced numbers of measurements (f). Maps were first estimated without fixing *T*1_*c*_ and *T*2_*c*_, using voxel-wise fitting (a) or slice-wise fitting (b: maps computed at the calibration stage). Second, *T*1_*c*_ and *T*2_*c*_ were fixed according to the literature (from [9] and [19] in c and d respectively), or to calibrated values (e, f). Maps are displayed in radiological convention. Histograms show the number of voxels with a given *f*_*my*_ difference (in %) between each *f*_*my*_ map and the reference map (a).

**Fig 3:**
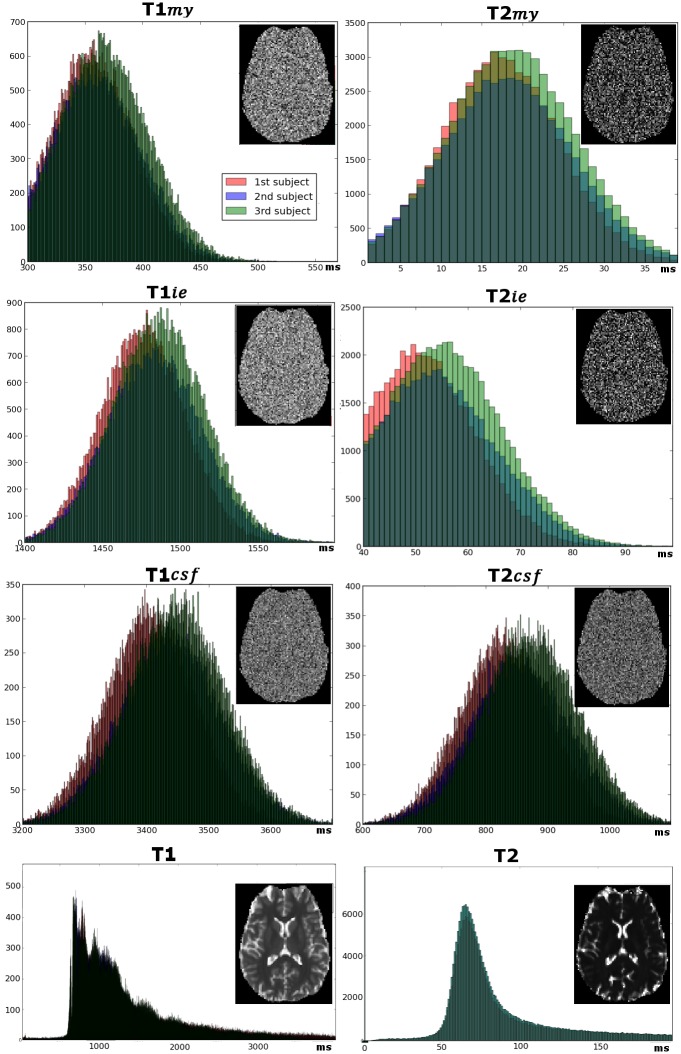
Variations of the compartments relaxation times across voxels. In the first three rows, histograms show distributions of *T*1_*c*_ and *T*2_*c*_ estimations (for myelin-related water, intra/extra-cellular water, and CSF compartments) obtained from voxel-wise fitting within the search ranges, across all voxels of the 3 calibration datasets. *T*1_*c*_ and *T*2_*c*_ maps (presented for subject #3) did not reveal any regional dependence. In the bottom row, distributions of quantitative T1 and T2 values obtained from mono-exponential fittings at the voxel-level are shown, as well as T1 and T2 maps (subject #3).

In the calibration datasets, the voxel-wise fitting procedure provided *f*_*my*_ maps of high quality (Fig 2a). Across voxels, *T*1_*c*_ and *T*2_*c*_ estimations varied to some extent over the whole search ranges, but in a completely random way (Fig 3), suggesting these magnetic properties can be considered as stable across brain regions. The resulting mean *T*1_*c*_ and *T*2_*c*_ values (±standard deviations SD) were:

- for myelin-related water compartment: *T*1_*my*_ = 360±39ms, *T*2_*my*_ = 18±8ms
- for intra/extra-cellular water compartment: *T*1_*ie*_ = 1482±29ms, *T*2_*ie*_ = 53±12ms
- for unrestricted water (CSF) compartment: *T*1_*csf*_ = 3431±78ms, *T*2_*csf*_ = 852±79ms.

In the same datasets, the calibration procedure resulted in the following *T*1_*c*_ and *T*2_*c*_ estimations (mean±SD over the 10 slices of the 3 subjects):

- for myelin-related water compartment: *T*1_*my*_ = 357 ± 21ms, *T*2_*my*_ = 18 ± 5ms
- for intra/extra-cellular water compartment: *T*1_*ie*_ = 1483 ± 17ms, *T*2_*ie*_ = 52 ± 6ms
- for unrestricted water (CSF) compartment: *T*1_*csf*_ = 3441 ± 36ms, *T*2_*csf*_ = 858 ± 47ms.

These values were close to the distribution peaks revealed by the voxel-wise procedure (no statistical difference: t-test, p>0.4). The resulting *f*_*my*_ maps (Fig 2b) had similar distributions of values across the 3 subjects (ad-hoc *χ*^2^ test, p>0.95), and similar visual appearances with voxel-wise *f*_*my*_ maps (Fig 2a), with differences smaller than 3% (histogram in Fig 2b). These results suggested that our slice-wise calibration procedure was successful in comparison with the voxel-wise procedure. On the contrary, fixing *T*1_*c*_ and *T*2_*c*_ according to the literature was inadequate as *f*_*my*_ maps displayed much smaller values than voxel-wise maps in most voxels, and were not consistent across subjects (Fig 2c and 2d). *f*_*my*_ values were very low in the grey matter, despite the known presence of myelin in both the cortex and deep grey nuclei. These observations confirmed that the calibration stage was necessary to estimate reliable *T*1_*c*_ and *T*2_*c*_ given our acquisition framework.

### Factors Influencing the Calibration Stage

#### Selection of *f*_*my*_ upper boundary

We investigated how the initial upper search boundary for *f*_*my*_ impacted the estimated *f*_*my*_, *T*1_*c*_ and *T*2_*c*_ values at the calibration stage. With a [0, 0.4] interval, quite high *f*_*my*_ values were observed in the adult white matter (Fig 2b), in comparison with previous studies [7,9]. When changing the upper boundary to 0.3 and 0.5 for the 3 calibration datasets, we observed a strong impact on *f*_*my*_ maps (S3 Fig): higher upper boundary resulted in higher *f*_*my*_ values in all 3 subjects. However, the estimated *T*1_*c*_ and *T*2_*c*_ were very close (S2 Table). Of interest, when the upper boundary was set to 0.4, distributions of *f*_*my*_ values across the 3 subjects were the most similar (i.e. they had the biggest overlap, S3 Fig), and standard deviations for *T*1_*c*_ and *T*2_*c*_ estimations were the lowest (S2 Table). These results suggested that fixing the upper boundary for *f*_*my*_ to 0.4 was valid although it might provide high *f*_*my*_ values.

#### Accuracy of the calibration strategy

We further assessed whether our calibration strategy was sensitive to different acquisition parameters (sampling points, noise level). First, reducing the number of calibration TI and TE points until N = 25 for both T1 and T2 relaxometry signals, had no significant effect on *T*1_*c*_ and *T*2_*c*_ calibration in subject #3 (ad-hoc paired t-test between *T*1_*c*_ and *T*2_*c*_ values from individual slices: p>0.06, S4 Fig). This suggested that our calibration stage was performed in a stable manner across all 3 subjects despite the different numbers of TI and TE points.

**Fig 4:**
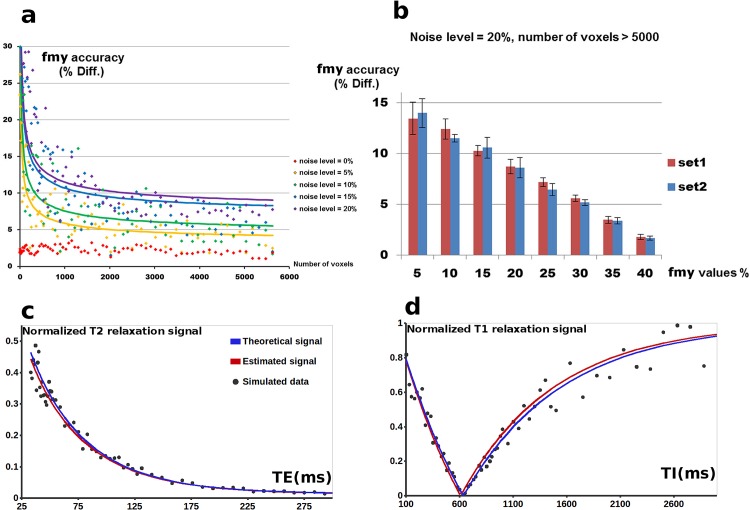
*f*_*my*_ calculation accuracy at the calibration stage assessed in numerical simulations. (a) The *f*_*my*_ calculation errors were calculated for simulated *f*_*my*_ = [5,10,15,20,25,30,40]% and *f*_*csf*_ = [0,1,2,3,4,5]%, and the resulting average values were represented as a function of the number of voxels used for the model fitting [1^2^, 2^2^, …, 75^2^ = 5625], for different noise levels (from 0 to 20%). As expected, relationship between the *f*_*my*_ calculation errors and the number of voxels could be described by inverse square root relationship (*R*^2^ > 0.75). (b) For a noise level of 20%, calculation errors for different simulated *f*_*my*_ values were averaged for voxel numbers higher than 5000 ([71^2^, …, 75^2^]) and *f*_*csf*_ = [0,1,2,3,4,5]%, considering *T*1_*c*_ and *T*2_*c*_ values fixed according to our calibration stage (set1) or to previous studies (set2 [9,19,34,35]). (c, d) Examples of the theoretical and estimated T2 (c) and T1 (d) relaxation signals for data simulated with *f*_*my*_ = 20%, *f*_*ie*_ = 78%, *f*_*csf*_ = 2% and *T*1_*c*_ and *T*2_*c*_ fixed according to our calibration stage.

Application of the calibration strategy to simulated data showed that as the noise level increased, reliable calculation of *f*_*my*_ values required increasing the number of voxels used to estimate the compartment relaxation times (Fig 4a). In agreement with previously conducted simulations [19], the average estimation errors were less than 10% even for a noise level of 20% of the signal values, on condition that more than 2000 voxels were used simultaneously (Fig 4a). In simulations with a 20% noise level and a number of voxels higher than 5000, we observed that estimation errors decreased with increasing *f*_*my*_ values, and that maximal errors did not exceed 15% for *f*_*my*_ values of 5% (Fig 4b). This suggested that the number of voxels considered in our calibration stage (~5718±406 voxels for each slice) was sufficient for reliable *f*_*my*_ estimation.

Besides, in identical conditions (same noise level, number of voxels, compartment fractions, *T*1_*c*_ and *T*2_*c*_), removing T1 relaxation from the model (i.e. removing Eq 3) significantly reduced the accuracy of *f*_*my*_ calculations as compared to the full model (ad-hoc paired t-test, *p* < 10^−6^). This demonstrated that modeling T1 and T2 relaxometry signals together was more appropriate than considering T2 relaxation alone, this was probably because we could not sample T2 signals for very short TE as compared with the very low *T*2_*my*_ value as discussed below.

### Evaluation of the Testing Stage

#### Fixing *T*1_*c*_ and *T*2_*c*_ with calibrated values

In the calibration datasets, fixing *T*1_*c*_ and *T*2_*c*_ with calibrated values to fit the 3-compartment model had little effect on *f*_*my*_ maps, whether the number of data points was kept complete (Fig 2e) or reduced to 8 TI and 8 TE values matching those of the reduced datasets (Fig 2f). The average absolute differences between the reference *f*_*my*_ maps and *f*_*my*_ maps computed with fixed calibrated *T*1_*c*_ and *T*2_*c*_ were very low (-0.3±1.8%; Fig 2e and 2f).

#### *f*_*my*_ maps in the reduced datasets

**Fig 5:**
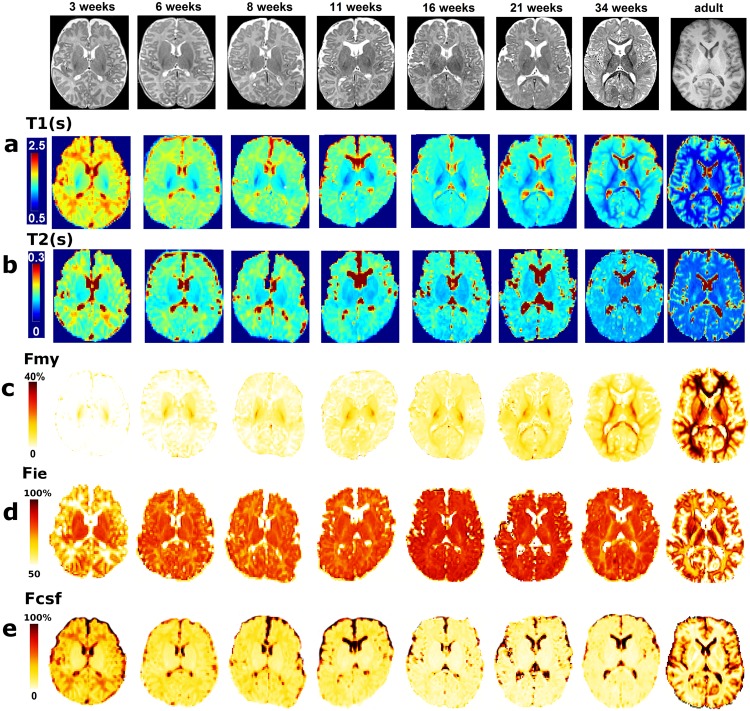
T1, T2 and fraction maps computed in reduced datasets. Quantitative T1 (a) and T2 (b) maps are presented for infants at different ages and for an adult subject, with corresponding anatomical images (T2w for infants / T1w for the adult). Axial slices are displayed in radiological convention. T1 and T2 maps show the expected decreases with age. Fraction maps for the compartments of myelin-related water *f*_*my*_ (c), intra/extra-cellular water *f*_*ie*_ (d) and unrestricted water *f*_*csf*_ (e) obtained at the testing stage are also presented. In agreement with the known age-related decrease in water content, *f*_*csf*_ decreased with age in most voxels except in the CSF, while *f*_*my*_ increased in white matter regions with myelination. Interestingly, the early increase in *f*_*ie*_ during infancy was followed by a decrease from 34 weeks on in locations where *f*_*my*_ showed concomitant sharp increase (i.e. posterior limb of the internal capsule and optic radiations).

In adults, *f*_*my*_ maps from the reduced datasets were very similar to the maps from the calibration datasets, both in terms of visual appearance (Figs 2 vs 5) and distribution of *f*_*my*_ values (ad-hoc *χ*^2^ test: p>0.95). In infants, *f*_*my*_ maps showed the myelination progression described by *post-mortem* studies [43]. Within white matter, the youngest infants had high *f*_*my*_ values only in the posterior limb of the internal capsule, and *f*_*my*_ values progressively increased with age in a caudo-rostral direction, from the center of the brain to the periphery (Fig 5).

### *f*_*my*_ Quantification in White Matter Bundles

#### *f*_*my*_ in the adult bundles

**Fig 6:**
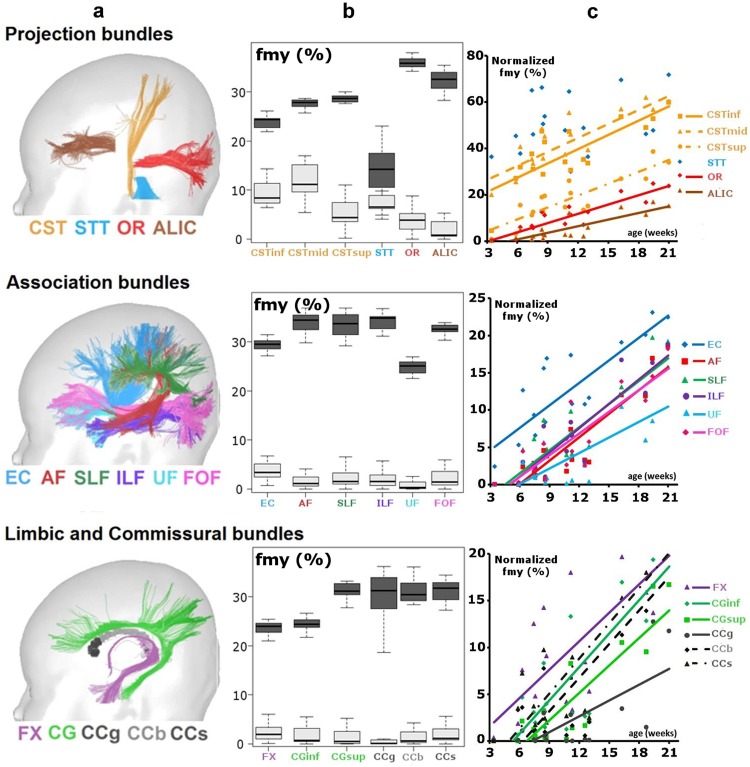
*f*_*my*_ quantification across white matter bundles in infants and adults. (a) White matter bundles reconstructed with tractography (adapted from [42]). (b) *f*_*my*_ values across bundles in infants (light) and adults (dark). (c) Age-related changes in infants’ normalized *f*_*my*_ values (in % of the mean adult values) for the same bundles. Lines show significant linear regressions with age (*R*^2^ > 0.58, p<0.015), for all bundles except the spino-thalamic tract. Abbreviations: *Projection bundles*: cortico-spinal tract CST (inf—inferior, mid—middle, sup—superior portions), spino-thalamic tract STT, optic radiations OR, anterior limb of the internal capsule ALIC; *Association bundles*: external capsule EC, arcuate fasciculus AF, superior SLF and inferior ILF longitudinal fascicles, uncinate fasciculus UF, fronto-occipital fasciculus FOF; *Limbic and Commissural bundles*: fornix FX, inferior CGinf and superior CGsup parts of the cingulum; corpus callosum: genu CCg, body CCb and splenium CCs.

In adults, *f*_*my*_ values were highly variable across bundles (Fig 6a and 6b): from 0.14 in the spino-thalamic tract up to 0.36 in the optic radiations. In the spino-thalamic tract, surprisingly low values and high inter-individual variability were probably due to subtle misalignment between *f*_*my*_ maps and DW images in the brainstem of some adults. High variability in *f*_*my*_ values was also observed in the genu of corpus callosum, possibly due to partial volume effects with the ventricle CSF. In all bundles, *f*_*my*_ values were strongly correlated with T1 values across adult subjects (*R*^2^ > 0.95), but not with T2 values.

#### *f*_*my*_ in the infant bundles

In infants, *f*_*my*_ values were smaller than in adults and below 0.05 for most bundles (on average over the group) (Fig 6b). The highest *f*_*my*_ values were observed in the cortico-spinal and spino-thalamic tracts and in the optic radiations, which are known to myelinate early on. According to the 3-compartment model, age-related changes in T1 and T2 relaxation times (measured at the voxel level) over the infant group could be explained by age-related changes in the compartment fractions, notably by changes in *f*_*my*_ values. For instance, in the middle cortico-spinal tract, we observed that *f*_*my*_ changed from 5% at 3 weeks of age to 17% at 21 weeks, in relation with changes in *f*_*ie*_ from 89% to 80%, and in *f*_*csf*_ from 6% to 3%. According to Eq 3 and calibrated *T*1_*c*_ and *T*2_*c*_, T1 was expected to change from 1321 to 979ms, in agreement with experimental observations (the measured T1 changed from 1322 to 978ms on average over the tract). These results were coherent with our initial hypothesis that age-related changes in relaxometry signals and T1 / T2 relaxation times (measured at the voxel level), could be modeled exclusively by age-related changes in the compartment fractions.

*f*_*my*_ values normalized by the mean values from the adult group increased with age (from 3 to 21 weeks) in all bundles but in an asynchronous manner (Fig 6c): in certain bundles (e.g. spino-thalamic tract, cortico-spinal tract, external capsule, fornix, optic radiations), normalized *f*_*my*_ values increased earlier than in other bundles (e.g. arcuate and uncinate fasciculi, anterior limb of the internal capsule, corpus callosum). In all bundles except the spino-thalamic tract, linear regressions could describe these age-related increases over this short developmental period. However, since certain bundles had zero *f*_*my*_ values in the youngest infants, non-linear fitting may be more pertinent for describing the earliest subtle changes in bundle myelination [29].

## Discussion

In this work we proposed an original 2-stage approach for fast *f*_*my*_ quantification, based on a 3-compartment model (myelin-related water, intra/extra-cellular water, unrestricted water) of T1 and T2 relaxometry signals. At the first stage, we calibrated the compartment inherent relaxation times (*T*1_*c*_ and *T*2_*c*_) using data acquired in healthy adult subjects with a long acquisition protocol. At the second stage, *f*_*my*_ maps could be computed using a simplified linear model and relaxometry data acquired with a short acquisition protocol more adequate for a pediatric population.

### Is This 2-Stage Strategy Valid to Quantify f_my_ in the Adult Brain?

#### Checking the measures of *f*_*my*_, *T*1_*c*_ and *T*2_*c*_

One may first wonder whether our main hypothesis (i.e. fixing the compartment relaxation times to identical values across all brain regions and tissues) is valid in the adult brain. Indeed, when we applied a voxel-wise fitting to the calibrating datasets, *T*1_*c*_ and *T*2_*c*_ did not show any regional dependence across voxels, and their distributions showed mean values close to those identified during calibration. Furthermore differences in *f*_*my*_ values across methods were rather low (<3% Fig 2a, 2b and 2e). Thus local variations in *T*1_*c*_ and *T*2_*c*_ were not expected to significantly vitiate our results in adults. However, these magnetic properties likely depend on acquisition protocol, and fixing their values based on the literature (Fig 2c and 2d) was less reliable than considering calibrated values. Similarly, in future studies with different acquisition settings, calibrating *T*1_*c*_ and *T*2_*c*_ values would be required rather than applying the values reported here.

At the calibration stage, the initial search ranges for *T*1_*c*_ and *T*2_*c*_ were selected based on literature evidence [9,19,34,35], and identified values were in agreement with reported values [6,9,33,44]. Before fixing *T*1_*c*_ and *T*2_*c*_ to compute *f*_*my*_ maps in the reduced datasets, we checked that their calibration was stable by 1) showing that all calibration datasets had enough sampling points for robust estimation of the compartment magnetic properties (S4 Fig); 2) verifying using simulated data that the proposed calibration procedure resulted in reliable estimations of *f*_*my*_ values even in the presence of noise in relaxometry signals (Fig 4a and 4b); 3) proving that fixing these relaxation times and down-sampling the calibration datasets did not change the generated *f*_*my*_ maps (Fig 2e and 2f). Experiments with simulated data further suggested that in the presence of noise, calculation of *f*_*my*_ values might not be reliable on a voxel-wise level, and that increasing the number of voxels used to calibrate *T*1_*c*_ and *T*2_*c*_ was then required (Fig 4a).

#### Comparison with other methods

With our approach, the calibration stage naturally required long acquisition (>35min) and post-processing times (up to 24 hours, without parallel computing). However, it must be performed only once for a given acquisition protocol (magnetic field, acquisition sequences, spatial resolution, etc.), and could be drastically accelerated using parallel computing. The main advantage of this approach is that, once calibration is performed, *f*_*my*_ values can be computed over the whole brain within much shorter acquisition (~5/7min for 50/70 slices with 1.8mm isotropic resolution, in infants/adults correspondingly) and post-processing times (<5min) than in previous studies. Indeed, in conventional multi-compartment relaxometry imaging studies [11], acquisition time takes up to 15 min for 8–12 contiguous slices because estimation of the T2 spectrum requires acquisition of the relaxometry data with a large number of TE. Although alternative approaches using mcDESPOT protocol [8] have enabled to shorten the acquisition time (between 18 and 25 min for a 1.8mm isotropic whole brain coverage on a 3T scanner, including B_0_ and B_1_ corrections [21], or around 10–12 min without these corrections [7]), these times remain quite long for unsedated infants, and rather long post-processing times (i.e. ~14 hours per subject [19]) are still required. Thus, using the proposed strategy can save at least 10 min of acquisition time and ~14 hours of post-processing time.

Our computed *f*_*my*_ values were higher than in most previous studies of infants [7,9] and adults [45], despite some exceptions [26,28]. Simulations suggested that our strategy was reliable even in the presence of noise in relaxometry signals. Nevertheless, local variations in the biophysical tissue properties and hence, in *T*1_*c*_ and *T*2_*c*_ across voxels might have introduced some additional “physiological” noise, leading to slight overestimation of *f*_*my*_ values (indeed, *f*_*my*_ values from the calibration stage were slightly higher than voxel-wise *f*_*my*_ values). It should also be noted that our model did not directly take into account the exchange between the different compartments: it only assumed that this exchange was fast relatively to T1 relaxation and slow relatively to T2 relaxation but it did not explicitly use exchange rate constants. Although these assumptions are generally true [9], ignoring exchange between compartments may also result in systematic bias [19,35]. However, including exchange through additional free parameters (exchange rate constants), would prevent the model to be linear, and our post-processing strategy would lose in reliability and speed. Similarly, our model neglected susceptibility differences across compartments, which might interfere particularly at high magnetic field, when these differences may impact the compartments relaxation. Correcting this would require to model a local susceptibility field gradient with a sinc function, but at the expense of an additional free parameter (i.e. the gradient amplitude). With fast-gradient echo sequences and in mcDESPOT protocols [21], not correcting for B0 and B1 field inhomogeneities might lead to errors in *f*_*my*_ calculations (e.g. overestimated *f*_*my*_ values [46]) since the signal is spatially dependent on the accuracy of the net flip angle. However, our protocol to measure T1 and T2 magnetic properties was based on spin echo EPI sequences, with repetition times much longer than T1 relaxation times, and MR signal was sampled for different TIs and TEs respectively. Thus inhomogeneities in B0 and B1 fields would impact images in the same way at all different TIs and TEs, and might corrupt proton density maps but without any impact on T1 and T2 estimations.

Eventually, our potential bias in high *f*_*my*_ values may arise from the stochastic fitting strategy used during calibration. Actually, in white matter structures, voxel-wise region contraction approach tend to assign *f*_*my*_ values to the upper part of the initial search interval, and the corresponding *T*2_*my*_ values to the lower part of the initial search interval [35]. Thus the initially selected search interval for *f*_*my*_ may have a considerable impact on *f*_*my*_ values. Indeed, we observed a strong impact on *f*_*my*_ maps when the upper boundary was changed from 0.4 to 0.3 or 0.5: higher boundary resulted in higher *f*_*my*_ values, and the 0.4 boundary provided the highest similarity in *f*_*my*_ distributions across the 3 subjects while the compartment relaxation times were stable. These observations point out to a main limitation of the present approach and possibly of other approaches using stochastic model fitting, meaning that *f*_*my*_ values strongly depend on the model fitting parameters. *f*_*my*_ quantification is also very sensitive to acquisition settings, including field strength, phase rewinding, sampling schemes of the inversion- and echo-times, voxel size, etc. [12,14,33,46]. This also justified the requirement to calibrate *T*1_*c*_ and *T*2_*c*_ instead of considering values from the literature. As a consequence, quantitative comparisons of *f*_*my*_ values across studies remain difficult.

#### Modeling both T1 and T2 relaxometry signals to measure *f*_*my*_

As myelin-related compartment has rather fast relaxation properties (e.g. *T*2_*my*_~20*ms*), robust estimation of its fraction from T2 spectrum requires to start TE sampling with small values [10–12]. In this study, single-shot EPI sequences were used to keep the acquisition time as short as possible; and such short TEs could not be achieved, mainly due to the further lines of the partial k-space located before the echo time [47]. This means that even at the shortest TE, T2 relaxometry signal from myelin-related water was already strongly degraded. Consequently, estimating *f*_*my*_ values from T2 spectral analysis only was not possible, and reliably fitting the 3-compartment model required adding information from T1 relaxation in order to reveal the contribution of myelin-water compartment. However, information from T2 relaxometry signal did help to separate intra-/extracellular water from unrestricted water compartments. Thus in such experimental conditions, considering both T1 and T2 relaxometry signals was necessary. In our study the SNRs were high and similar for both T1 and T2 relaxation images; thus, to balance T1 and T2 contributions to the model fitting error, the weight of Eq 3, describing T1 relaxation, was increased proportionally to the number of TI points. However, it may be necessary to adjust the relative weights of the model equations if SNRs differ between T1 and T2 relaxation signals. These different issues might explain at least in part why *f*_*my*_ and T1 values were strongly correlated in all adult bundles, similarly to previous studies using mcDESPOT strategy [45], in addition to the dramatic influence on T1 values of water compartmentalization and of macromolecules (large lipids and proteins) in the myelin sheath.

### Is This Approach Valid for Quantifying f_my_ in the Developing or Pathological Brain?

#### Estimation of f_my_ in the infant brain from T1_c_ and T2_c_ calibration in the adult brain

In addition to the verified hypothesis on compartmental property stability across adult brain tissues and regions, we also assumed stability throughout development, enabling us to compute *f*_*my*_ maps in infants using calibrated *T*1_*c*_ and *T*2_*c*_ in adults. Actually the hypothesis that age-related changes in T1 and T2 relaxation signals measured at the voxel level could be explained exclusively by changes in the compartment fractions within voxels, was verified in our infant group.

This assumption of compartment properties stability is often implicitly used in *f*_*my*_ studies [9,48] as well as in multi-compartment diffusion imaging studies [49]. Nevertheless, it might be questioned as compartments might change their biophysical properties during development, thus possibly affecting their relaxation times. For example, does the increase in myelin compactness with maturation shorten *T*1_*my*_ and *T*2_*my*_ values of the myelin-related compartment? Interestingly, the chemical composition of myelin in the infant brain was shown to be similar to that of the adult brain [50], meaning that *T*1_*my*_ and *T*2_*my*_ should be identical across different ages. Similarly, do *T*1_*ie*_ and *T*2_*ie*_ values of the intra-/extra-cellular compartment change with the development of intra-cellular cytoskeleton and microtubules?

Anyhow, investigating possible age-related changes in the compartments relaxation times was beyond the scope of this study for two main reasons: healthy infants cannot withstand long acquisition protocols without sedation, and maturation is not homogeneous across brain regions [43,51,52]. First, to be coherent with the issue raised previously, the estimation of *T*1_*c*_ and *T*2_*c*_ at the calibration stage should be performed independently for regions with different maturational levels. This would require voxel-wise (and not slice-wise) fitting at the individual level, which is hardly achievable because of acquisition time. Estimating these magnetic properties is not realistic at the group level either, i.e. using data from different infants: even at the same age, inter-individual differences in brain development are observed, and it would be a major challenge to register tissues with strictly similar microstructure and maturation. Second, even if calibration of maturation-specific *T*1_*c*_ and *T*2_*c*_ was realistic, it would not have much sense at the testing stage. To fit the model within a single infant brain, different *T*1_*c*_ and *T*2_*c*_ would be needed for different regions with different maturations. Such an approach would require the maturation level to be known *a priori* in each voxel, creating a vicious circle. To summarize, only a voxel-wise estimation of *f*_*my*_, *T*1_*c*_ and *T*2_*c*_ would be entirely free from model hypotheses, and considering exchange between compartments would be helpful. Nevertheless these aspects might be hardly achievable in healthy infants. Despite possible limitations related to the assumption of *T*1_*c*_ and *T*2_*c*_ stability across brain regions and throughout development, our approach based on calibration and testing stages remains a reliable and realistic strategy in such populations.

As detailed in S1 Supporting Information, we observed a strong correlation between the age-matched infant *f*_*my*_ maps that we obtained and those computed by another group [8,29]. These latter studies used an alternative approach based on age-range optimized mcDESPOT protocols and the voxel-wise 3-component fitting of relaxometry signals. Some differences in *f*_*my*_ values were observed across these and our studies. They might rely either on the use of adult *T*1_*c*_ and *T*2_*c*_ to fit our model equations in infants, or on the fact that mcDESPOT *f*_*my*_ estimates might represent not only the magnetization associated with the water pool trapped between the myelin sheaths, but also the magnetization transfer from non-aqueous myelin protons [35]. Thus comparing our results with previous studies across all ages remains difficult [8]. Furthermore, the authors did not indicate whether *T*1_*c*_ and *T*2_*c*_ were stable across the life span. Some correlations between *f*_*my*_ values, T1 and T2 (computed at the voxel level) were observed in infants. As both relaxation times do not exclusively reflect white matter myelination and as they change according to different maturational processes [5], their correlations with *f*_*my*_ values are likely to be age-dependent [8].

#### Modeling 3 compartments to measure f_my_

In our model, the number of model compartments was set to 3 because it has been shown to be adequate under a wide range of different conditions, including in the developing white matter [9,19,29,53,54]. Although it is possible to quantify *f*_*my*_ based on 2 compartments, 3-compartment models are thought to be more reliable, especially in regions with partial volume [19]. Furthermore, consistently with a 3-compartment model, T2 spectrum in both *in vitro* and *in vivo* experiments has shown three major peaks [22,39,55–57]. If necessary, our model could be easily extended with additional compartments, at the expense of increased model fitting complexity due to additional free-parameters. However, theoretical considerations suggest that there is no scope for deriving more than 2 or 3 components in healthy tissues [58].

The most reliable approach to estimate *f*_*my*_ without assumptions on the number of compartments and on their magnetic properties, might be to calculate T2 spectrum [10,11]. However in this case reliable estimation would require acquisition of relaxometry signals for a large number of TEs (making acquisition time unacceptably long for infants), including very short TEs that were not achievable with EPI sequences. Recent acquisition sequences with ultra-short TE (UTE) might be helpful in the future to sample the T2-weighted signal over a wider range of TEs while keeping a short acquisition time. Simulations further suggested that in the presence of noise, using a 3-compartment model with fixed *T1*_*c*_ and *T2*_*c*_ could significantly improve accuracy and reproducibility in determining compartments fractions, as compared to T2 spectrum [22].

#### Potential applications under pathological conditions

Although 3-compartment models have been previously applied to investigate pathologies [19,24,53,54,59,60], such as multiple sclerosis [60], autism [53] and partial deletions of chromosome 18q [54], one should be careful when trying to apply the suggested approach to diseases. Indeed it might require including additional compartments (e.g., microvascular, tumor, inflammatory cells, gliosis, etc.) to account for pathological tissues [33]. For example, in demyelinating diseases, gliotic tissue (tissue matrix that replaces myelin, being filled with water, sparse cells and macromolecules) might be modeled as a separate compartment; if not, it would likely contribute to the intra-/extracellular compartment, which has the most similar properties to gliotic tissue among the 3 compartments. Consequently, applying the suggested approach to pathological conditions would require taking into account possible additional compartments specific to the pathology, and re-calibrating the compartment relaxation times in patients.

In conclusion, our *f*_*my*_ quantification strategy overcame existing difficulties (long acquisition/post-processing times) that might limit its practical application in infants, children and clinical patients. Although reliable comparison with previous studies [7,8] was not achievable, our infant *f*_*my*_ maps were able to capture myelin-related changes across early development, suggesting that the proposed approach was relevant at least for evaluation of normal maturation.

## Supporting Information

S1 Fig TI and TE sampling in the adult datasets. a: For the 3 calibration datasets, 30 to 60 TI values, and 47 to 60 TE values (plotted on a log scale) were used to acquire T1 and T2 relaxometry signals. Different TI/TE ranges were sampled with different steps, and higher sampling of low TI and TE values was performed. See also S1 Table. b: The initial full dataset for subject #3 contained 60 TI and TE data points. Reduced datasets (plotted on a log scale) were obtained by progressively reducing the number of data points in a regular manner from 60 down to 10 points, i.e. by removing/keeping every n^th^ point from the initial dataset so as to have the desirable number of data points (e.g. we kept every 2^nd^ sampling point to have a reduced dataset of 30 points, every 12th point to have a reduced dataset of 5 points…). These reduced datasets were used to investigate whether the number of TI and TE sampling points impacted the estimation of *T*1_*c*_ and *T*2_*c*_.
(TIF)

S2 Fig Convergence of the voxel-wise model fitting algorithm. a: The length of the search intervals for *T*1_*c*_ and *T*2_*c*_ (between the upper and lower boundaries) is illustrated as a function of time (in % from the initial total length, blue curve) in a typical voxel within white matter. The convergence required around 3 repetitions of the fitting procedure: each repetition is indicated with brackets, containing 10000 random solutions and taking around 25s. At the end of each repetition (arrows), the search intervals were contracted by selecting the best 100 solutions (over 10000) of *T*1_*c*_ and *T*2_*c*_ samples. For the first 90% samples of the next repetition (over 10000), *T*1_*c*_ and *T*2_*c*_ values were randomly chosen from the contracted intervals. Boosts at the end of the second and third repetitions (at around 50 and 75s) corresponded to the remaining 10% samples when *T*1_*c*_ and *T*2_*c*_ values were randomly chosen from the initial non-contracted intervals to avoid falling into a local minimum. Note that after the first contraction, the search intervals already met the stopping criteria (length less than 1% of the initial search intervals).
For each sample of *T*1_*c*_ and *T*2_*c*_, we also presented (red curve) the length of the intervals corresponding to the *on-going* best 100 solutions considering *all previous T*1_*c*_ and *T*2_*c*_ samples. This length quickly decreases to 0, explaining the dramatic interval contraction at the end of the first repetition. Besides, it is not strictly monotonic because at each step a better solution might appear outside the previous interval, particularly at the beginning of the fitting procedure. b: Residuals of the model fitting (Eq 1–4) are presented for the same voxel as in a. After the first repetition and interval contraction, most solutions were quite close to the acceptance level (fitting error less than 1%), which was met after two further small contractions. This graph suggests that relaxing the stopping criteria might be possible to decrease the computation time without losing much precision.
Similar convergence was observed in voxels of grey matter and CSF (fitting not shown).
(TIF)

S3 Fig Comparison of *f*_*my*_ maps generated with different upper search *f*_*my*_ boundaries. For the 3 calibration subjects, *f*_*my*_ maps generated at the calibration stage for different *f*_*my*_ upper boundary of 0.3 (a), 0.4 (b) and 0.5 (c) show differences in *f*_*my*_ amplitudes: higher boundary led to higher *f*_*my*_ values, although the estimated *T*1_*c*_ and *T*2_*c*_ were similar (S2 Table). In the right column, histograms show *f*_*my*_ distributions across all voxels of the 3 subjects. The percentages of common histogram area across the 3 subjects (82%, 85%, 79% for *f*_*my*_ of 0.3, 0.4 and 0.5 respectively) suggested that the upper boundary of 0.4 had the biggest overlap, and thus, the highest reproducibility across subjects.
(TIF)

S4 Fig Impact of the number of calibration data points on the estimation of *T*1_*c*_ and *T*2_*c*_. For subject #3, *T*1_*c*_ and *T*2_*c*_ values of the 3-compartment model (mean ± standard deviation over the 10 central slices) were calculated with the calibration strategy using various numbers N of TI and TE sampling points (S1 Fig). When this number was higher than 25, *T*1_*c*_ and *T*2_*c*_ values did not significantly differ from those calculated with N = 60 (ad-hoc paired t-test between the values from individual slices, * indicates significant difference where p<0.05).
(TIF)

S1 Table TI and TE sampling in the adult calibration datasets. For each calibration subject, the sampling schemes of TI and TE points (values and numbers) are detailed, as well as the total acquisition time. For example, for Subject #1, three sets of TI values and three sets of TE values were used, covering different ranges with different steps in order to provide higher sampling of low TI and TE values. Note that schemes differed across subjects, and that sets might present overlapping ranges but different sampling values (e.g. TI sets for subject #3). See also S1 Fig.
(DOCX)

S2 Table *T*1_*c*_ and *T*2_*c*_ calibrated for different *f*_*my*_ upper search boundary. Mean and standard deviations are computed over the 10 slices of the 3 subjects after the calibration stage. Note that *T*1_*c*_ and *T*2_*c*_ values are roughly the same, while standard deviations tend to be the lowest for the 0.4 upper boundary.
(DOCX)

S1 Supporting Information Comparison of *f*_*my*_ maps obtained in infants with our approach and with mcDESPOT sequences. The infant *f*_*my*_ maps were voxel-wise correlated with the age-matched *f*_*my*_ maps computed by another group from data acquired using mcDESPOT sequences [25] (http://www.babyimaginglab.com/Research_files/meanMWFMaps.zip). For that, infants’ anatomical T2w images were co-registered using affine transformations to the 3D Pediatric T1w templates corresponding to the age-matched *f*_*my*_ maps. The resulting transformations were applied to both infants’ T2w images and *f*_*my*_ maps, and registered images were correlated voxel-wise with the age-matched *f*_*my*_ maps. Strong voxel-wise correlations (*R*^2^ > 0.77) were observed for our *f*_*my*_ maps, and they were significantly higher (p<0.001, ad-hoc paired t-test) than for infants’ anatomical T2w images (*R*^2^ > 0.67). This suggested high similarity in *f*_*my*_ values, which could not be explained simply by similarity in underlying anatomical structures.
(DOCX)
