# Supplementary figures and images for: A New Strategy for Fast MRI-Based Quantification of the Myelin Water Fraction: Application to Brain Imaging in Infants
Source: PLoS One. 2016 Oct 13;11(10):e0163143. doi: 10.1371/journal.pone.0163143 (PMC5063462; doi:10.1371/journal.pone.0163143)

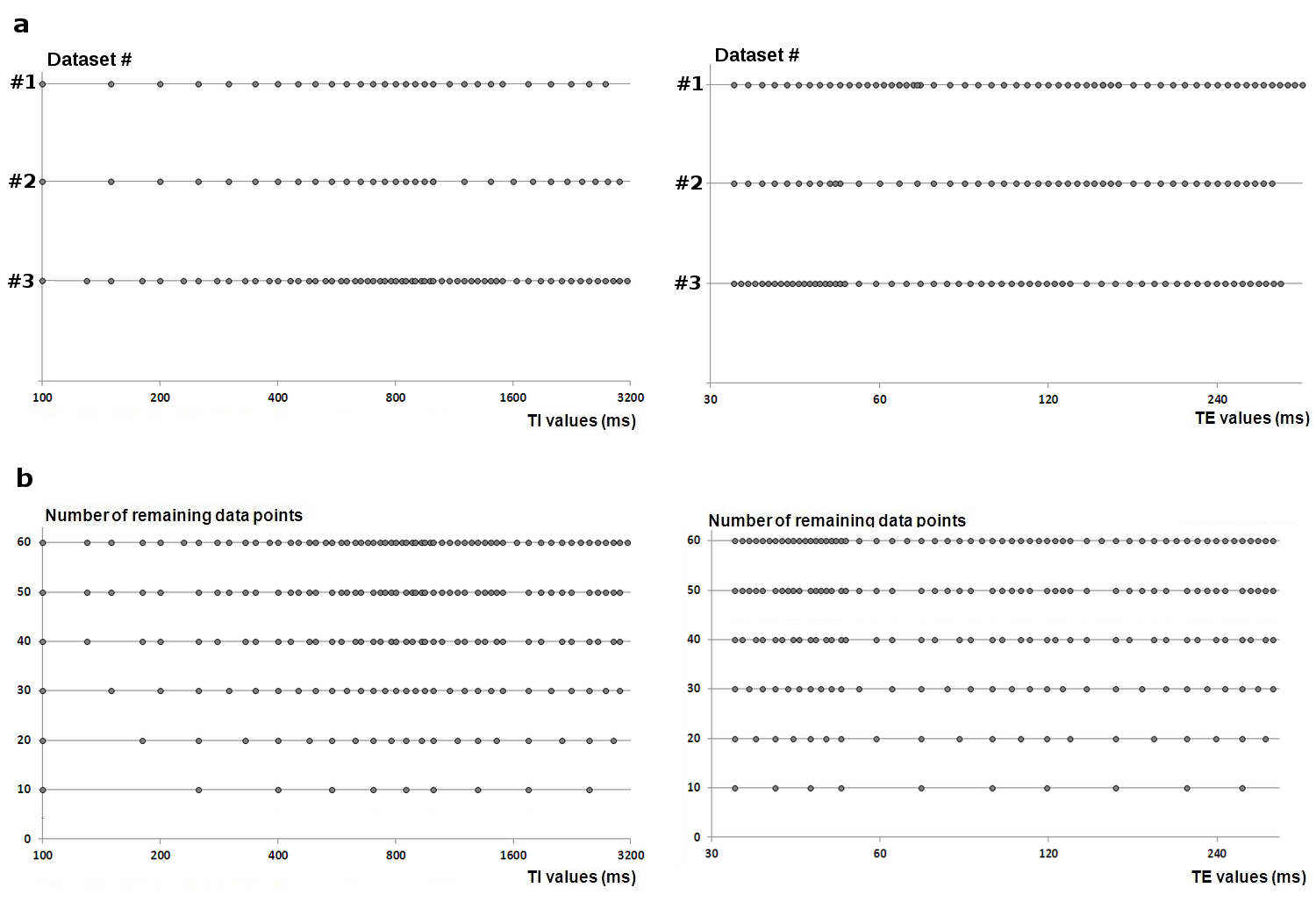

Supplement: S1 Fig — a: For the 3 calibration datasets, 30 to 60 TI values, and 47 to 60 TE values (plotted on a log scale) were used to acquire T1 and T2 relaxometry signals. Different TI/TE ranges were sampled with different steps, and higher sampling of low TI and TE values was performed. See also S1 Table. b: The initial full dataset for subject #3 contained 60 TI and TE data points. Reduced datasets (plotted on a log scale) were obtained by progressively reducing the number of data points in a regular manner from 60 down to 10 points, i.e. by removing/keeping every nth point from the initial dataset so as to have the desirable number of data points (e.g. we kept every 2nd sampling point to have a reduced dataset of 30 points, every 12th point to have a reduced dataset of 5 points…). These reduced datasets were used to investigate whether the number of TI and TE sampling points impacted the estimation of T1c and T2c. (TIF) [file pone.0163143.s001.tif]

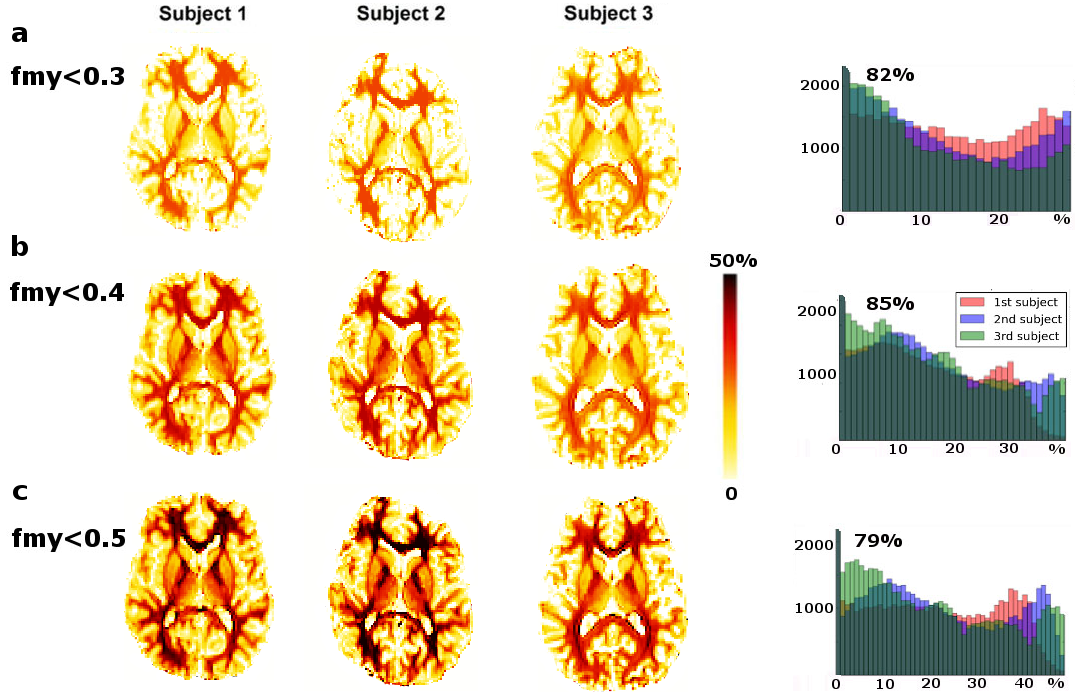

Supplement: S3 Fig — For the 3 calibration subjects, fmy maps generated at the calibration stage for different fmy upper boundary of 0.3 (a), 0.4 (b) and 0.5 (c) show differences in fmy amplitudes: higher boundary led to higher fmy values, although the estimated T1c and T2c were similar (S2 Table). In the right column, histograms show fmy distributions across all voxels of the 3 subjects. The percentages of common histogram area across the 3 subjects (82%, 85%, 79% for fmy of 0.3, 0.4 and 0.5 respectively) suggested that the upper boundary of 0.4 had the biggest overlap, and thus, the highest reproducibility across subjects. (TIF) [file pone.0163143.s003.tif]

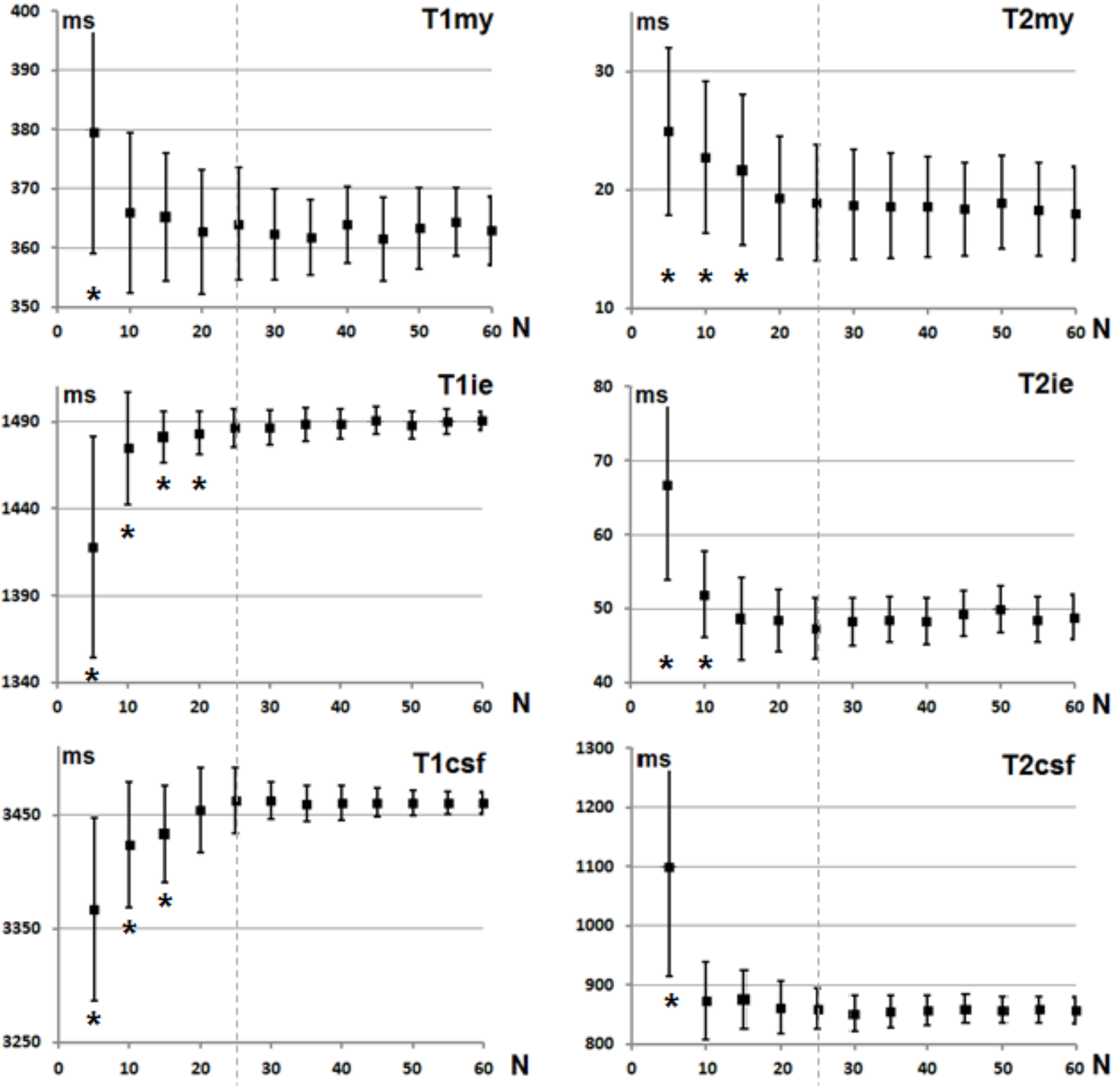

Supplement: S4 Fig — For subject #3, T1c and T2c values of the 3-compartment model (mean ± standard deviation over the 10 central slices) were calculated with the calibration strategy using various numbers N of TI and TE sampling points (S1 Fig). When this number was higher than 25, T1c and T2c values did not significantly differ from those calculated with N = 60 (ad-hoc paired t-test between the values from individual slices, * indicates significant difference where p<0.05). (TIF) [file pone.0163143.s004.tif]
