# Supplementary material for: A New Strategy for Fast MRI-Based Quantification of the Myelin Water Fraction: Application to Brain Imaging in Infants
Source: PLoS One. 2016 Oct 13;11(10):e0163143. doi: 10.1371/journal.pone.0163143 (PMC5063462; doi:10.1371/journal.pone.0163143)
Supplement: S1 Table — For each calibration subject, the sampling schemes of TI and TE points (values and numbers) are detailed, as well as the total acquisition time. For example, for Subject #1, three sets of TI values and three sets of TE values were used, covering different ranges with different steps in order to provide higher sampling of low TI and TE values. Note that schemes differed across subjects, and that sets might present overlapping ranges but different sampling values (e.g. TI sets for subject #3). See also S1 Fig. (DOCX) [file pone.0163143.s005.docx]

**S1 Table: TI and TE sampling in the adult calibration datasets.**

For each calibration subject, the sampling schemes of TI and TE points (values and numbers) are detailed, as well as the total acquisition time. For example, for Subject #1, three sets of TI values and three sets of TE values were used, covering different ranges with different steps in order to provide higher sampling of low TI and TE values. Note that schemes differed across subjects, and that sets might present overlapping ranges but different sampling values (e.g. TI sets for subject #3). See also S1 Fig.

| *Subject*  *(acq. time)* | *TI values* | | | | | *TE values* | | | | |
| --- | --- | --- | --- | --- | --- | --- | --- | --- | --- | --- |
|  | *low border (ms)* | *upper border (ms)* | *step (ms)* | *Number of points* | | *low border (ms)* | *upper border (ms)* | *step (ms)* | *Number of points* | |
| *Subject 1*  *(41min 30s )* | *100* | *1000* | *50* | *19* | *30* | *33* | *71* | *2* | *20* | *60* |
|  | *1100* | *1500* | *100* | *5* |  | *65* | *160* | *5* | *20* |  |
|  | *1750* | *3000* | *250* | *6* |  | *150* | *340* | *10* | *20* |  |
| *Subject 2*  *(34min 54s)* | *100* | *1000* | *50* | *19* | *30* | *33* | *51* | *2* | *10* | *47* |
|  | *1000* | *3000* | *200* | *11* |  | *50* | *160* | *5* | *23* |  |
|  |  | | | |  | *170* | *300* | *10* | *14* |  |
| *Subject 3*  *(47min 11s)* | *100* | *1500* | *50* | *29* | *60* | *33* | *52* | *1* | *20* | *60* |
|  | *130* | *980* | *50* | *18* |  | *55* | *131* | *4* | *20* |  |
|  | *1630* | *3130* | *250* | *7* |  | *140* | *311* | *9* | *20* |  |
|  | *1750* | *3000* | *250* | *6* |  |  |  |  |  |  |
