# Supplementary material for: A New Strategy for Fast MRI-Based Quantification of the Myelin Water Fraction: Application to Brain Imaging in Infants
Source: PLoS One. 2016 Oct 13;11(10):e0163143. doi: 10.1371/journal.pone.0163143 (PMC5063462; doi:10.1371/journal.pone.0163143)
Supplement: S2 Table — Mean and standard deviations are computed over the 10 slices of the 3 subjects after the calibration stage. Note that T1c and T2c values are roughly the same, while standard deviations tend to be the lowest for the 0.4 upper boundary. (DOCX) [file pone.0163143.s006.docx]

**S2 Table: *T1_c_* and *T2_c_* calibrated for different *f_my_* upper search boundary.**

Mean and standard deviations are computed over the 10 slices of the 3 subjects after the calibration stage. Note that *T1_c_* and *T2_c_* values are roughly the same, while standard deviations tend to be the lowest for the 0.4 upper boundary.

|  | $f_{my}<0.3$ | $f_{my}<0.4$ | $f_{my}<0.5$ |
| --- | --- | --- | --- |
| ${T2}_{my}$*(ms)* | *21±10* | *18±5* | *18±4* |
| ${T2}_{ie}$*(ms)* | *59±9* | *52±6* | *48±6* |
| ${T2}_{csf}$*(ms)* | *885±49* | *858±47* | *848±62* |
| ${T1}_{my}$*(ms)* | *359±16* | *357±21* | *321±21* |
| ${T1}_{ie}$*(ms)* | *1325±150* | *1481±17* | *1426±46* |
| ${T1}_{csf}$*(ms)* | *3203±210* | *3441±36* | *3403±75* |
