# Supplementary material for: A New Strategy for Fast MRI-Based Quantification of the Myelin Water Fraction: Application to Brain Imaging in Infants
Source: PLoS One. 2016 Oct 13;11(10):e0163143. doi: 10.1371/journal.pone.0163143 (PMC5063462; doi:10.1371/journal.pone.0163143)
Supplement: S1 Supporting Information — The infant fmy maps were voxel-wise correlated with the age-matched fmy maps computed by another group from data acquired using mcDESPOT sequences [25] (http://www.babyimaginglab.com/Research_files/meanMWFMaps.zip). For that, infants’ anatomical T2w images were co-registered using affine transformations to the 3D Pediatric T1w templates corresponding to the age-matched fmy maps. The resulting transformations were applied to both infants’ T2w images and fmy maps, and registered images were correlated voxel-wise with the age-matched fmy maps. Strong voxel-wise correlations (R2 > 0.77) were observed for our fmy maps, and they were significantly higher (p<0.001, ad-hoc paired t-test) than for infants’ anatomical T2w images (R2 > 0.67). This suggested high similarity in fmy values, which could not be explained simply by similarity in underlying anatomical structures. (DOCX) [file pone.0163143.s007.docx]

**S1 Supporting Information. Comparison of** $\boldsymbol{f}_{\boldsymbol{my}}$ **maps obtained in infants with our approach and with mcDESPOT sequences.**

The infant $f_{my}$ maps were voxel-wise correlated with the age-matched $f_{my}$ maps computed by another group from data acquired using mcDESPOT sequences [25] (<http://www.babyimaginglab.com/Research_files/meanMWFMaps.zip>). For that, infants’ anatomical T2w images were co-registered using affine transformations to the 3D Pediatric T1w templates corresponding to the age-matched $f_{my}$ maps. The resulting transformations were applied to both infants’ T2w images and $f_{my}$ maps, and registered images were correlated voxel-wise with the age-matched $f_{my}$ maps. Strong voxel-wise correlations ($R^{2}>0.77$) were observed for our $f_{my}$ maps, and they were significantly higher (p<0.001, ad-hoc paired t-test) than for infants’ anatomical T2w images ($R^{2}>0.67$). This suggested high similarity in $f_{my}$ values, which could not be explained simply by similarity in underlying anatomical structures.
